# SenolyticSynergy: An Attention-Based Network for Discovering Novel Senolytic Combinations via Human Aging Genomics

**DOI:** 10.3390/ijms26189004

**Published:** 2025-09-16

**Authors:** Yaowen Ye, Ting Su, Jiayi Gao, Dengming Ming

**Affiliations:** College of Biotechnology and Pharmaceutical Engineering, Nanjing Tech University, Nanjing 211816, China; yyw121379@gmail.com (Y.Y.); supermarysu@outlook.com (T.S.); 2020230604@stu.cpu.edu.cn (J.G.)

**Keywords:** senolytics, aging genome, drug combinations, anti-aging interventions

## Abstract

Senolytics, a category of drugs targeting aging processes, have garnered significant attention since their emergence in 2015. Unlike traditional drug development approaches that rely on randomized screening, research on aging-related pharmaceuticals has employed mechanism-based strategies, resulting in the discovery of the pioneering combination therapy of dasatinib (D) and quercetin (Q). Although preliminary studies with senolytic drug combinations have shown promising outcomes, the predictive capabilities of the research in this field remain limited by the extensive experimental data requirements. In this study, we employed differential gene expression analysis and machine learning techniques to investigate the combinatorial effects of senolytic drugs. We identified 1624 core aging-related genes and used this dataset to retrain a multimodal attention mechanism model, creating a specialized framework, SenolyticSynergy, for predicting effective senolytic drug combinations. We then utilized 63 established senolytic compounds as starting points for combination testing, developing a comprehensive dataset of 1953 potential drug combinations for aging interventions. Following rigorous filtration, we identified 190 high-confidence drug combinations and predicted their synergistic scores. Among these combinations, ten demonstrated exceptionally high synergistic scores, exceeding 8. The combination of temsirolimus and nitazoxanide ranked first and may be the most promising candidate. The analysis of the literature data and computational studies of molecular structures using 3D modeling validated the accuracy of these predictions. This framework paves the way for large-scale research into anti-aging drug combinations, advancing research capabilities in this field.

## 1. Introduction

In recent years, the scientific community is proposed to redefine aging as a chronic disease [1]. Its pathological trajectory is intertwined with the mechanisms of malignant tumors [2], the pathological development of neurodegenerative diseases and many other pathological processes. Within this macro-physiological process, the biological homeostasis of cells and tissues is affected, leading to a gradual decline in cellular function and genomic stability. Together, these two factors increase the risk of cancer and neurodegenerative diseases as we age. Several theories attempt to explain the molecular mechanisms underlying aging. The oxidative stress theory suggests that the gradual accumulation of reactive oxygen species (ROS) damages DNA, proteins, and lipids, impairing cellular function. The telomere shortening theory posits that progressive loss of telomeric DNA during cell divisions leads to replicative senescence. The DNA damage and genomic instability theory emphasizes the accumulation of mutations and impaired DNA repair as drivers of cellular decline. However, according to all those theories, senescent cells are always the primary targets for intervention. Senescent cells remain metabolically active but no longer divide and typically secrete pro-inflammatory and tissue-remodeling factors, known as the senescence-associated secretory phenotype (SASP). Cellular senescence is a state of permanent cell-cycle arrest that normal cells enter in response to stresses such as telomere shortening, DNA damage, or oncogenic signaling. Senolytics are a diverse class of drugs specifically targeting aging cells for clearance. By combining senolytic drugs, it is possible to delay and inhibit aging-related behaviors at various cellular, tissue, and organ levels, ultimately intervening in the aging process of individuals and promoting healthy aging.

In the past decade, numerous machine learning-based models have been developed to predict drug combination synergy. For instance, Zhao and colleagues proposed the Dual Feature Fusion Network for Drug–Drug Synergy prediction (DFFNDDS), which utilizes a fine-tuned pre-trained language model and dual feature fusion mechanism to predict synergistic drug combinations [3]. Wang and colleagues introduced EDDINet4, which enhances drug–drug [4] interaction (DDI) prediction via Information Flow and Consensus-Constrained Multi-Graph Contrastive Learning for precise DDI prediction. Li and colleagues built a two-view deep learning model, JointSyn [5], for predicting the synergistic effects of drug combinations and applied it to identify drug combinations for pan-cancer. Monem and colleagues proposed MultiComb [6], a multi-task deep learning (MTDL) model designed to predict the synergy and sensitivity of drug combinations simultaneously. This model creatively utilizes a graph convolutional network to represent the Simplified Molecular-Input Line-Entry (SMILES) of two drugs, generating their respective features. Guo and colleagues introduced SynergyX, a multi-modality mutual attention network designed to improve anti-tumor drug synergy prediction [7], which dynamically captures cross-modal interactions, enabling the modeling of complex biological networks and drug interactions. Yan and colleagues proposed DconnC (Drug-molecule Connect Cell) and GTextSyn, the former leveraging cellular features as nodes to establish connections between drug molecular structures, allowing the extraction of pertinent features [8]. The latter utilized the integration of gene expression data and chemical structure information to create sentences with biochemical relational significance for the prediction of synergistic effects in drug combinations on Cance [9]. Sun and colleagues brought out GraphTranSynergy for accurate drug synergy prediction, which showed significant advantages in biological interpretability via Graph Transformer and BiLSTM (Bidirectional Long Short-Term Memory) [10].

In this study, we utilized various R (Version 4.2.2) packages and methods [11] to analyze and process GSE141595 [12] and GSE72815 [13], accurately identifying significantly differentially expressed genes (DEGs) between the two groups. Subsequently, we adopted the attention-based mechanism network. We integrated the identified aging-related DEGs as a targeted subset for model training, focusing on developing a drug combination prediction model for aging-related diseases to achieve a precise prediction of synergistic effects between drugs.

We successfully identified multiple core differential genes related to aging through differential analysis, including *PKP1*, *NRAP*, and *CMA1*. For model fitting pre-screening, we employed an approach that involved the initial screening and subsequent calculation of 1953 drug combinations formed by 63 senolytics discovered and experimentally validated by 1 July 2024. After the screening, 193 valid entries were retained for drug combination synergy scoring. Ultimately, we retrained the prediction model to obtain ten drug combinations, including temsirolimus + nitazocine and temsirolimus + fiserone, which displayed exceptionally high synergistic scores in the model evaluation (predicted synergy score > 8), indicating significant therapeutic effects in aging interventions. Drugs with synergy scores exceeding eight were selected for literature validation.

Clustering analysis of differentially expressed genes has highlighted the significance of aging-related core pathways, such as hsa04020, demonstrating a robust theoretical foundation for a deeper understanding of the pathological mechanisms underlying aging diseases. Furthermore, published experimental studies have confirmed the synergistic effects of several drug combinations predicted by this model. For example, a survey by Ren [14] indicated that the combination of Cantharidin + ABT-737, among others, significantly improved the clearance of aging cells while maintaining low toxicity to normal cells. This discovery expands the horizons of anti-aging treatment strategies. This validates the feasibility of machine learning in discovering drug combinations, offering a more promising therapeutic outlook than single-drug treatments.

This study aimed to analyze the differential gene expression profiles between young and elderly populations and identify aging-related genes. These genes serve as key input elements for the drug-gene attention model, which predicts and optimizes the potential synergistic benefits of different anti-aging drug combinations. This study provides scientific evidence in support of precision medicine.

## 2. Results and Discussion

### 2.1. Differential Genetic Analysis

**Figure 1:**
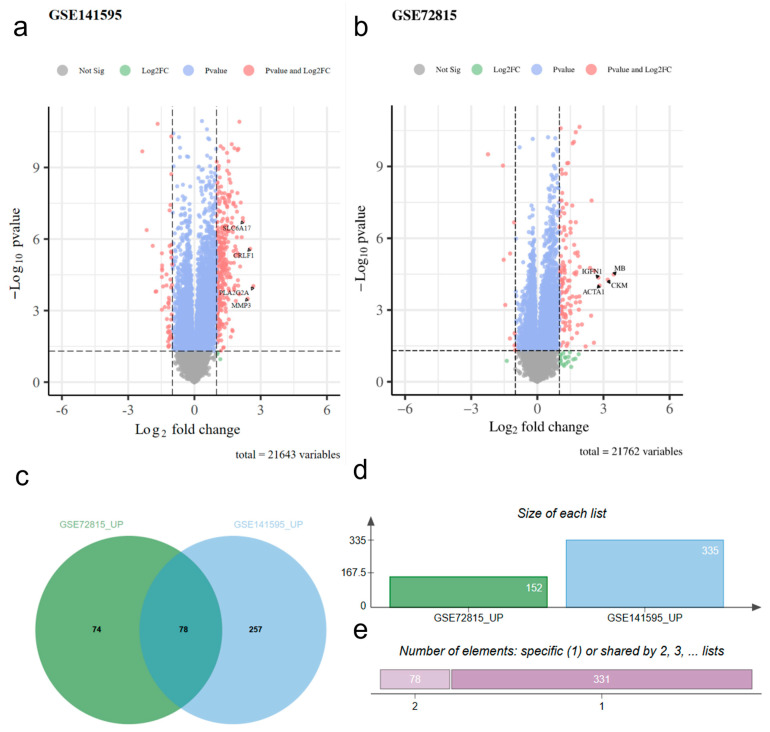
Aging Genome Analysis plots (**a**,**b**) illustrating the differentially expressed genes in the GSE141595 and GSE72815 datasets. The horizontal axis represents the log2 fold change, and the vertical axis represents the −log10 *p*-value. Each point represents a gene, with color coding and labels denoting the gene’s significance based on thresholds of fold change and *p*-value. In GSE141595, the significantly upregulated genes included *SLC6A17*, *CRLF1*, *PLAG2A*, and *MMP3*. Similarly, in GSE72815, the significantly upregulated genes included *MB*, *CKM*, *IGFN1*, and *ACTA1*. (**c**) displays the overlap of upregulated genes between the two datasets, revealing 78 genes commonly upregulated in the intersection of GSE141595 and GSE72815. This significant overlap mutually validates the reliability of the differential analysis data for the two gene sets. (**d**,**e**) present the total number of upregulated genes in the two datasets, amounting to 409 after excluding duplicate entries.

We obtained the gene expression data files of the GSE141595 and GSE72815 datasets by accessing the GEO (Gene Expression Omnibus) database and conducting differential gene expression analysis, using R package (Version 4.2.2) and DESeq2 program (Version 1.40.2) [15]. We applied a |log2FC| greater than 1.0 and a Padjust value less than 0.05 as filtering criteria, and the outcomes are shown in Figure 1. Furthermore, we identified the core genes by performing an intersection analysis between GSE141595 and GSE72815 (Table 1).

The genes listed in Table 1 have demonstrated high relevance to aging. For illustrative purposes, we selected five genes for discussion. *H19* [16] is a long non-coding RNA (lncRNA) that regulates gene expression and cellular aging. *HIF3A* encodes hypoxia-inducible factor 3α, a key regulatory factor in the cellular response to low-oxygen environments relevant to aging and lifespan regulation [17]. The *TREM2* gene is associated with neurodegenerative diseases, such as Alzheimer’s disease, and influences the aging process of the brain [18]. *SERPINA3*, which encodes α1-antichymotrypsin, is associated with inflammation and aging [19]. Lastly, *ADAMTS12* encodes a protease involved in extracellular matrix remodeling associated with tissue aging and repair [20].

### 2.2. Enrichment Analysis

**Figure 2:**
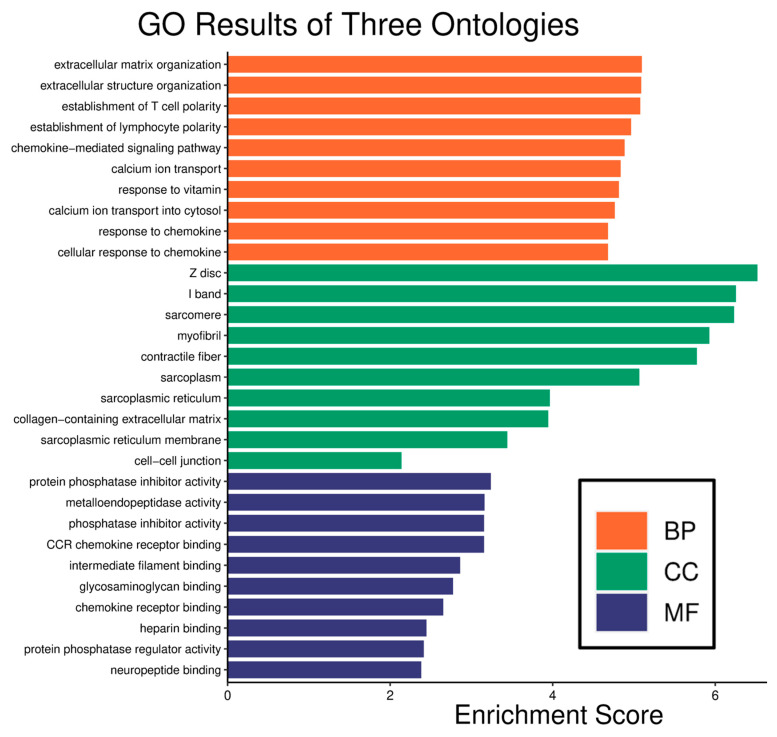
Senescence-related Differential Gene Enrichment Analysis. BP: The enrichment analysis of biological processes of aging-related core DEGs demonstrated their predominant involvement in extracellular matrix organization and extracellular structure organization. CC: The enrichment analysis of cellular components of aging-related core DEGs revealed a concentration of these genes in the Z disk, I band, and sarcomere, confirming their origin from bone tissue cells. MF: The enrichment analysis of molecular functions emphasized protein phosphatase inhibitor activity, metalloendopeptidase activity, phosphatase inhibitor activity, and CCR chemokine receptor binding, all closely associated with the aging process.

Enrichment analysis is a critical method in bioinformatics, primarily employed to elucidate the functional tendencies of gene sets within specific biological contexts [21]. GO (Gene Ontology) enrichment analysis utilizes the GO database for functional annotation and enrichment analysis of given gene sets. The GO database categorizes genes into three primary annotation categories: Biological Process (BP), Cellular Component (CC), and Molecular Function (MF) (Figure 2).

### 2.3. Pathway Analysis

The KEGG (Kyoto Encyclopedia of Genes and Genomes) pathway enrichment analysis is a significant bioinformatics method [22] for investigating genes’ involvement in specific biological processes and their participation in complex pathways, including cellular metabolism and signal transduction. This study utilized the R packages clusterProfiler and pathview to perform KEGG pathway enrichment analysis of the extracted core aging-related genes.

The table illustrates pathways involving genes that occur two or more times. There are 11 significant signaling pathways, including the calcium signaling pathway, which concerns the transmission of signals by calcium ions within cells. Calcium ions play crucial roles in organisms, including cell growth, differentiation, apoptosis, and muscle contraction.

Most of the signaling pathways presented in this table are intricately linked to aging. For instance, hsa04020, which ranks highest in relevance, involves calcium ions as vital cellular messengers in various biological functions, including cell proliferation, differentiation, programmed cell death, and muscular contractions. This pathway comprises two primary components: (1) External calcium ion sources: Cells obtain calcium ions from their surroundings through diverse calcium channels located on the cellular membrane, such as voltage- operated channels (VOCs), receptor- operated channels (ROCs), and store-operated channels (SOCs). (2) Internal calcium ion sources: The endoplasmic reticulum/sarcoplasmic reticulum (ER/SR) functions as a critical calcium ion storage site, where inositol 1,4,5-trisphosphate receptors (IP3Rs) and ryanodine receptors (RYRs) control the release of calcium ions.

Furthermore, the core pathways in Table 2 include proteins and signaling factors highly relevant to cancer regulation, such as the p53 and Wnt signaling pathways within the MAPK signaling pathway (hsa04010). The Wnt signaling pathway is critical for embryonic development, tissue repair, and cancer, influencing cell behavior by regulating proliferation, differentiation, migration, and stemness maintenance [23]. Abnormal activation of the Wnt pathway is associated with various cancer types, including colorectal and liver cancers, with mutations in tumor suppressor genes in the Wnt pathway (such as APC and AXIN2) leading to overactivation of β-catenin and promoting the growth and self-renewal of tumor cells.

### 2.4. Synergy Prediction and Interpretation

The prediction model we employed is called SenolyticSynergy. In this study, we integrated the dataset of omic genes 1624 genes to features and made the model specifically for senolytic target utilization. The following Table 3 presents the prediction results sorted by the prediction synergy score. (The entire table is available in Supplementary Materials).

Among the candidate drugs listed, several are FDA-approved for specific clinical indications. Temsirolimus (Torisel) is approved for advanced renal cell carcinoma (FDA approval: 30 May 2007). Nitazoxanide (Alinia) is approved for the treatment of diarrhea caused by Giardia lamblia in patients aged 12 years and older (FDA approval: 21 July 2004). Azithromycin (Zithromax) is approved for various bacterial infections (FDA approval: 1 November 1991). Cantharidin (Ycanth) is approved as a topical treatment for molluscum contagiosum in patients aged 2 years and older (FDA approval: 21 July 2023). Enoxacin was previously approved for various infections but has been withdrawn from the U.S. market and is no longer available.

By searching databases such as PubChem and DrugBank [26], we identified the pathways associated with various drugs to understand the principles underlying drug combinations, thereby enhancing the interpretability of the prediction results. Here are examples of two types of drug combinations with different benefits:

Combination with synergistic effects on the same pathway: For instance, the combination of Temsirolimus and Nitazoxanide. Both drugs inhibit the mTOR protein, thereby jointly blocking the PI3K-Akt signaling pathway, which corresponds to the pathways enriched by differential gene analysis of LAMA3/MET.

Combination with synergistic effects on different pathways, for example, the combination of Fisetin and Azithromycin. Azithromycin reduces inflammation by inhibiting the NF-κB signaling pathway, corresponding to the NF-kappa B signaling pathway. Conversely, Fisetin upregulates HO-1 expression via the p38 MAPK pathway, inhibiting doxorubicin-induced senescence of pulmonary artery endothelial cells. It also inhibits the proliferation of pulmonary artery smooth muscle cells through the Nrf2/HO-1 signaling pathway, thereby preventing pulmonary artery remodeling. These correspond to the MAPK and Nrf2/HO-1 signaling pathways.

The specific relationships between the individual drugs and enriched pathways are detailed in Table 4.

### 2.5. Verification

The inherent lack of interpretability in deep learning consistently poses challenges for model mechanism inference. In this study, we employed molecular docking simulations to validate the mechanisms of high-synergy drug combinations, enhancing the interpretability of synergistic effects in drug combinations and further elucidating the advantages of dual-drug systems. To validate the efficacy of our model, we conducted a literature review for the top ten predicted drug combinations. Based on the model’s scoring system, we identified a novel senolytic drug combination, Temsirolimus + Nitazoxanide, with the highest score. We performed molecular docking simulations to validate the interaction further and confirm its synergistic potential.

### 2.6. Molecular Docking Verification

We simulated the docking of the highest-scoring drug combination predicted by the model: Temsirolimus + Nitazoxanide on the same target. The docking results indicated that the binding sites of these two compounds on the mTOR target were highly similar. In the context of the dynamic structure of the three-dimensional conformation of the protein, this drug combination demonstrates a broader binding range for FKBP12 in mTORC1 compared to Temsirolimus alone. Temsirolimus is a prodrug of rapamycin that is rapidly converted to its active form by cytochrome CYP 4503A4/5 in the bone marrow. It exhibits superior chemical stability and solubility compared with rapamycin. Its active component, rapamycin, is lipophilic and can permeate the cell membrane to bind to the intracellular receptor FKBP-12 (FK506-binding protein 12 kDa) [44]. The resulting complex then binds to the FRB domain of the TOR protein, inhibiting its function.

We performed the molecular docking simulations to verify the binding modes of Temsirolimus and Nitazoxanide with the mTOR protein. The structures of the Temsirolimus and Nitazoxanide compounds were extracted from PDB codes: 7SQ9 and 3V35, respectively. The mTOR protein structure was obtained from PDB code: 7PED. The protein was pre-processed using AutoDock Tools (ADT) by removing water molecules and adding hydrogen atoms.

**Figure 3:**
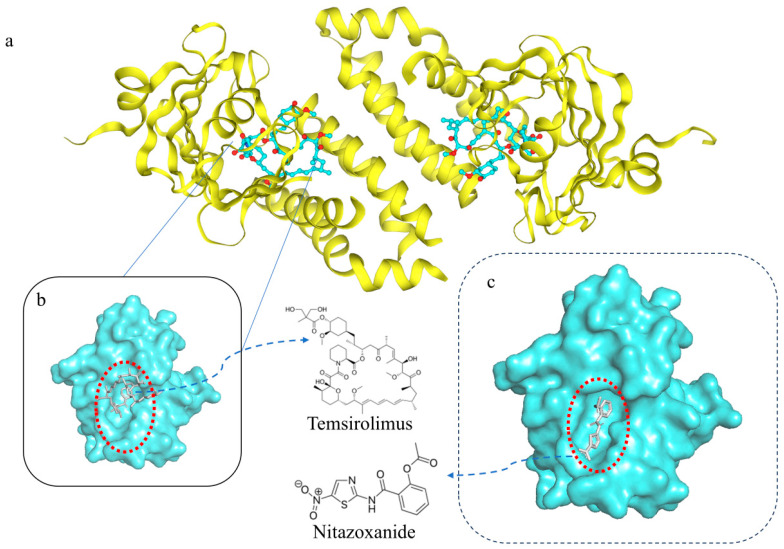
Molecular docking simulation verification of Temsirolimus and Nitazoxanide with the mTOR protein. (**a**) shows the mTOR protein’s original docking site with the Temsirolimus’s active component, (**b**) shows an enlarged view of the binding site between the active component of Temsirolimus and the mTOR protein, and (**c**) displays the binding site after docking nitazoxanide to the mTOR protein.

Simulated docking of the drugs with mTOR protein was performed using AutoDock Vina (v1.2.5) [45] and the MDPA pocket search program [46], revealing a high consistency in the target action region for this drug combination. Upon adjusting the three-dimensional docking view of both compounds to the same angle as in Figure 3b, it becomes evident that the binding regions of the individual drugs within this combination highly overlap with that of mTOR. From the perspective of three-dimensional dynamic protein complementarity, this effectively demonstrates the expansion of the target hit range following drug combination therapy. Table 5 presents the affinity of the molecule binding to the target protein under different modes after docking nitazoxanide to mTOR. (with Grid center (unit = Å): (50.524, −17.028, 15.563), Grid size: X = 43.443, Y = 43.851, Z = 53.900, and Grid space: 0.375).

The root-mean-square deviation (RMSD) measures the relative conformational distance from the most favorable mode. A more negative binding affinity indicates a stronger and more stable interaction between the drug and the receptor. Among the docking results, mode 1 demonstrated the lowest binding affinity (−7.259 kcal/mol), signifying the most optimal binding conformation. Consequently, mode 1 was selected as the reference mode, with its RMSD lower and upper bound values set to zero.

For other docking modes, modes 2–3 and 5–10 exhibited relatively high RMSD values (14.52 Å–23.67 Å) compared to the reference mode, suggesting significant spatial deviations from the optimal conformation and, consequently, lower reference significance. In contrast, mode 4 displayed an RMSD range of 3.092 Å–8.357 Å, indicating structural similarity to the best mode, with a corresponding binding affinity of −6.494 kcal/mol. The binding free energies for docking modes 1 and 4 fell within the range of −7.259 kcal/mol to −6.494 kcal/mol, suggesting that the binding conformation of nitazoxanide with mTOR at a certain binding site (as illustrated in Figure 3c) is relatively stable. Temsirolimus is a macrolide compound with a predominantly hydrophobic backbone, and hydrophobic interactions primarily drive its binding. In contrast, Nitazoxanide is a smaller molecule that contains multiple polar groups, such as an amide bond, nitro group, and thiazole ring, which can readily form hydrogen bonds or electrostatic interactions with polar or charged amino acid residues in the protein mTOR. This further elucidates the effective mechanism of action of the drug combination.

### 2.7. Literature Verification

A comprehensive literature review revealed that certain combinations listed in Table 3 have been experimentally validated. For the combination of Cantharidin and Fisetin, which has a predicted synergistic score of 10.35, Frezzato and colleagues [24] substantiated its joint action in inhibiting the binding of HSF1 to the HSP70 promoter, resulting in the downregulation of HSP70 expression.

Similarly, the combined treatment of Canthardin and ABT-737, with a predicted synergistic score of 9.70, was explored by Ren and colleagues [14], who empirically demonstrated its enhanced inhibitory effect on cell proliferation, targeting Bcl-2 family proteins relevant to aging. Na and colleagues [47] further confirmed, through in vitro experiments, the synergistic induction of apoptosis in cervical cancer cells with the combined application of norcantharidin (NCTD) and ABT-737, where NCTD significantly augmented the effect of ABT-737. Empirical evidence from the literature also highlighted the structural analog of baicalein, norcantharidin, in combination with ABT-737, elucidating a mechanism involving the inhibition of Mcl-1 through transcriptional suppression by NCTD, ultimately enhancing ABT-737-induced apoptosis in liver cancer cells. Norcantharidin and Cantharidin share similarities in chemical structure. Both compounds contain multiple cyclic structures and functional groups. Specifically, they both contain seven-membered and five-membered rings, which are connected by a carbonyl group. Additionally, both compounds contain two carboxyl groups in similar positions on the ring.

The specific action mechanisms involve NCTD promoting the mitochondrial translocation of Parkin, leading to changes in the mitochondrial membrane potential and an increase in mitochondrial reactive oxygen species (ROS), as well as inducing the accumulation of autophagic vacuoles and blocking autophagic flux, thereby regulating the expression of apoptosis-related proteins.

The combination of Finasteride and Azithromycin has been shown to have a predicted synergistic score of 9.64. Shao and colleagues [25] also confirmed that the combination of fisetin, a member of the flavonoid family, and azithromycin exhibits a more potent anti-inflammatory effect. The specific mechanism involves inhibiting phosphorylation in the JAK/STAT and MAPK pathways, as well as the nuclear translocation of NF-κB p65, thereby alleviating tubal factor infertility (TFI). Notably, fisetin is homologous to quercetin, with a molecular difference of a hydroxyl group at the C5 position.

## 3. Materials and Methods

### 3.1. Youth-Old Age Differential Gene Expression Dataset

Microarray data for this study were retrieved from the National Center for Biotechnology Information (NCBI) with accession numbers GSE72815 and GSE141595. In the GSE72815 study, hematopoietic stem cells and osteoblasts obtained through iliac crest needle biopsies were extracted from 58 healthy women, including 19 in the young women group (mean age ± standard deviation: 30.3 ± 5.4 years), 19 in the elderly women group (73.1 ± 6.6 years), and 20 older women (70.5 ± 5.2 years) who received 3 weeks of estrogen (E) treatment. Based on widely accepted criteria (false discovery rate [q] < 0.10), aging influenced a total of 678 genes and 12 pathways, including a subset of genes known to regulate bone metabolism [13].

GSE141595 involves gene profiling of trabecular bone biopsies from postmenopausal women who received either a placebo or denosumab treatment for 3 months, as well as gene analysis in young women who did not undergo treatment. We specifically utilized data from two groups: postmenopausal women receiving a placebo and young women not undergoing treatment [12].

### 3.2. Senolytics Dataset

We obtained part of our single-drug dataset from Smer-Barreto [48], then added the newest and found senolytics as supplementary to make the final table as follows:

Senolytic in Table 6 activity in vitro varies by drug class, with cardiac glycosides (digoxin, ouabain, bufalin) active at 5–100 nM, BCL-2 inhibitors (navitoclax, venetoclax, ABT-737) at 0.1–1 μM, and HSP90 inhibitors (geldanamycin, 17-AAG, 17-DMAG, XL888) at 0.1–2 μM. Dasatinib exhibits activity at 50–250 nM, while flavonoids such as quercetin, fisetin, and luteolin are effective at 5–20 μM. Other compounds, including piperlongumine, macrolides, statins, rapalogs, and chemotherapeutics, show senolytic effects within 0.1 nM to 10 μM, depending on the cell type and context. Experimental probes identified in discovery screens generally act at 0.5–5 μM.

### 3.3. Aging-Related Target Gene Dataset

The target genes were selected from the following six sources:

(1) From the differential gene analysis of GSE72815, 152 entries with logFC > 1 and 11 entries with logFC < −1 were selected.
(2) From the differential gene analysis of GSE141595, 335 entries with logFC > 1 and 90 with logFC < −1 were selected.
(3) The Human Ageing Genomic Resources (HAGR) database [64], specifically the GenAge resource package (https://www.genomics.senescence.info/genes/index.html (accessed on 4 September 2024)), was used to download the latest stable version of human aging-related genes (https://www.genomics.senescence.info/genes/human_genes.zip (accessed on 4 September 2024)). A total of 307 gene entries were extracted from GenAge and designated as Source Three in the tables, named GenAge_human.
(4) The latest stable version of the LongevityMap [65] (https://www.genomics.senescence.info/longevity/ (accessed on 4 September 2024)) was obtained from the LongevityMap (https://www.genomics.senescence.info/longevity/longevity_genes.zip (accessed on 4 September 2024)), reflecting the current understanding of human longevity genetics. However, this database includes records of negative results; therefore, only 273 gene entries with “significant” status in the Association column were selected and recorded in the longevityMap table.
(5) A list of genes associated with cellular senescence was obtained from the CellAge database26 (https://genomics.senescence.info/cells/ (accessed on 4 September 2024)), which focuses on cellular senescence genes (https://genomics.senescence.info/cells/cellAge.zip (accessed on 4 September 2024)). Entries under the “Unclear” attribute of the Senescence Effect were excluded, and the remaining 927 records were extracted and recorded in the table.
(6) From the Aging Atlas database [66], aging-related gene sets were selected for download within the Aging-related gene sets section, resulting in 503 entries recorded in the Aging Atlas table.

**Figure 4:**
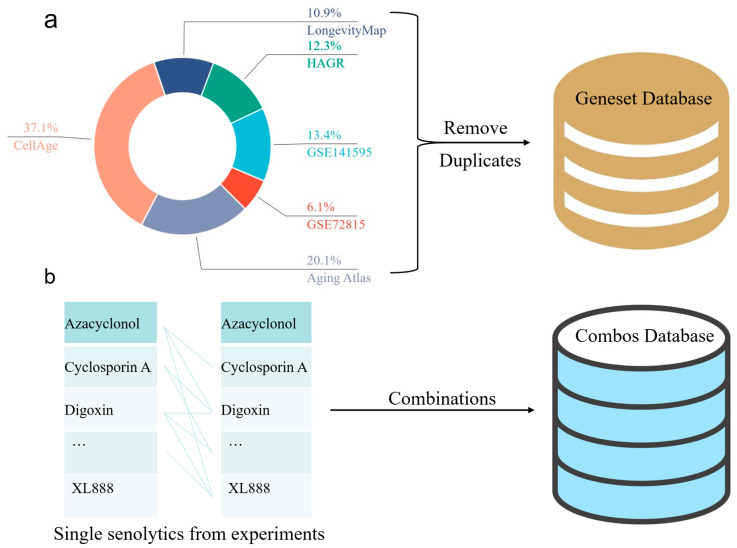
Database Components. (**a**) This pie chart depicts the distribution of different databases in the entire dataset. The most significant proportion is occupied by “CellAge” at 37.4%, followed by “Aging Atlas” at 20.1%, “GSE147591” at 13.4%, “Longevity Map” at 10.9%, “HAGR” at 12.3%, and “GSE72815” at 6.1%. After preprocessing, a database of 1624 unique gene entries was obtained by removing duplicates from the remaining data. (**b**) The left column presents the drugs used in individual experiments, such as Cyclosporin A and Digoxin. The drugs were combined pairwise to create a new database, resulting in 1953 unique drug combinations.

The total number of gene entries under these six approaches was 2566. After removing duplicate entries, 1769 entries remained for analysis. Finally, gene ID conversion was performed, and entries that could not be converted were removed, resulting in the final selection of 1624 target genes (Figure 4a) and 1953 drug combinations (Figure 4b).

### 3.4. Senolytics Combination Efficacy Prediction Model

**Figure 5:**
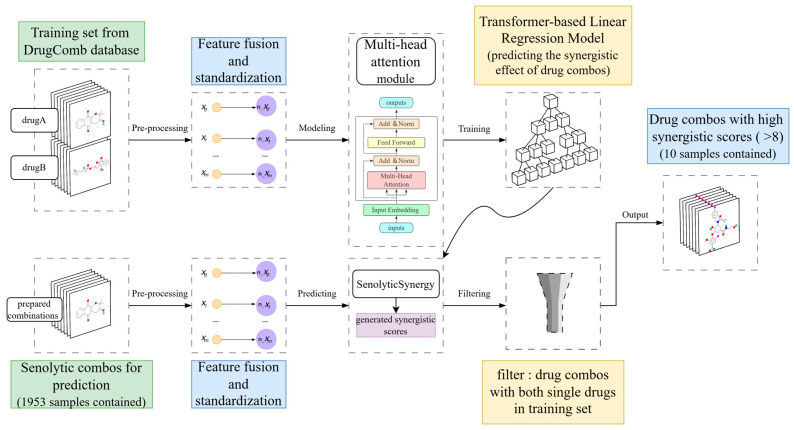
The overview of SenolyticSynergy.

Regarding model prediction, we introduced SenolyticSynergy (Figure 5), a classical attention-based [67] mechanism network designed to enhance the prediction of the synergistic effects of senolytic drugs. This model utilizes a classic attention structure that effectively integrates multiple omics data within its framework.

The training set collects 330,917 drug combinations from the DrugComb database consisting of 354 single drugs and 170 cell lines with S synergy scores [68], and performs drug-target gene feature fusion and feature standardization during preprocessing, builds the model with the multi-attention mechanism and inputs the features for training, and selects the optimal parameters as our prediction model: SenolyticSynergy. Here, we extracted binary drug-target gene features for both drugs and gene expression profiles for the corresponding cell line. These features were concatenated into a single input vector and standardized via Z-score normalization. The fused vectors were then input into a multi-head attention module consisting of an input embedding layer, two multi-head self-attention blocks (each with eight attention heads), and a feedforward neural network with two hidden layers, including 256 and 128 neurons, respectively, with ReLU activation and dropout (rate = 0.3) applied for regularization. The model was trained using the Adam optimizer (learning rate = 0.003, batch size = 128) for up to 100 epochs with early stopping (patience = 10), and optimized using mean squared error (MSE) loss.

A 1953 senolytic combinations used for prediction were also subjected to feature fusion and standardization, and the corresponding synergy scores of each combination were predicted by the SenolyticSynergy model. Meanwhile, we further filtered out 190 cases of senolytic combinations in which all single drugs appeared in the training set. We selected those combinations with synergy scores greater than 8 as the final output, based on the theory of Lin et al. that the training set contained single drugs within the prediction set that improved the accuracy of synergy prediction of drug combinations (40% to 90%) [69].

## 4. Conclusions

This study, which uses differential genes associated with aging as the core entry point, thoroughly investigates the intricate relationships between senolytic drugs and apoptosis-related pathways. By integrating multi-omics analysis with advanced deep learning models, we have successfully applied a multi-drug combination prediction model that specifically evaluates the efficacy of senescent cell clearance. Based on this robust foundation, we have constructed a high-confidence senolytic drug combination database for predictive purposes, led by the combination of temsirolimus and nitazoxanide. The predicted results have been partially validated through extensive experimental literature data and molecular docking simulation calculations. This work provides a novel perspective on the diversity of clinical drug use, offers a potential solution to the challenge of limited combinatorial therapies in the clinical application of senolytic drugs, and may provide valuable insights into personalized aging prevention strategies.

Since the analytical workflow in this study involved performing differential expression analysis separately on GSE72815 and GSE141595, and then extracting the intersection of DEGs, datasets with the same sex were specifically selected to avoid the loss of core aging-related genes due to sex-related differences. Nonetheless, it is acknowledged that this approach introduces a limitation with respect to sex. We supplemented this approach by incorporating multiple aging-related gene databases to compensate for this limitation.

Further experimental validation must take into account additional clinical considerations, such as dosage optimization and potential toxicity arising from drug combinations. We hope that research teams with qualifications for conducting human clinical trials will take particular interest in the top three ranked drug combinations identified in this study. Regarding future model improvements, graph-based approaches and deep ensemble learning techniques may be explored to enhance predictive performance further.

## Figures and Tables

**Table 1:** Common DEGs from GSE141595 and GSE72815.

Co-Expressed Differential Genes	Gene Name
Upregulated genes	*IGFN1*, *PTCHD4*, *PKP1*, *NRAP*, *CMA1*, *MIR675*, *AQP7B*, *ADCYAP1R1*, *ANGPTL7*, *MYOZ1*, *HOTS*, *LOC112267876*, *JPH2*, *LOC102724852*, *PLN*, *H19*, *LINC01436*, *MUC3A*, *MYH1*, *LMO3*, *PLCXD3*, *HIF3A*, *ADAMTS15*, *SRL*, *CASQ2*, *HOXD9*, *REM1*, *HSPB6*, *MEOX1*, *NTM*, *SLC52A3*, *CCL21*, *PTN*, *CYP26B1*, *INSRR*, *IGHV7-4-1*, *ADAMTSL1*, *COX4I2*, *LAMA3*, *GPR15*, *SERPINA3*, *FLNC*, *PERM1*, *TLL1*, *TREM2*, *STXBP6*, *NES*, *CCDC85A*, *LOC105375249*, *RERGL*, *CLIC5*, *SH3RF2*, *SYPL2*, *CCL19*, *RASD2*, *TCF15*, *CACNA1H*, *SLCO2A1*, *ALDH1A2*, *SSTR1*, *C1QTNF7*, *GPR17*, *KRT222*, *POSTN*, *FAM107A*, *PLPPR4*, *L1CAM*, *ANKRD29*, *TRIM63*, *IRX6*, *STC1*, *LOC105377979*, *MET*, *SHISAL1*, *TCEAL2*, *EBF2*, *ADAMTS12*, *SLIT3*
Downregulated genes	*PTPRQ*, *CRH*

**Table 2:** Enrichment pathway list of aging genome DEGs.

ID	Description	Gene ID	Count
hsa04020	Calcium signaling pathway	*PLN/CASQ2/CACNA1H/MET*	4
hsa04360	Axon guidance	*L1CAM/MET/SLIT3*	3
hsa04510	Focal adhesion	*LAMA3/FLNC/MET*	3
hsa05415	Diabetic cardiomyopathy	*CMA1/PLN/COX4I2*	3
hsa04024	cAMP signaling pathway	*ADCYAP1R1/PLN/SSTR1*	3
hsa04010	MAPK signaling pathway	*FLNC/CACNA1H/MET*	3
hsa00830	Retinol metabolism	*CYP26B1/ALDH1A2*	2
hsa04260	Cardiac muscle contraction	*CASQ2/COX4I2*	2
hsa04713	Circadian entrainment	*ADCYAP1R1/CACNA1H*	2
hsa04061	Viral protein interaction with cytokine and cytokine receptor	*CCL21/CCL19*	2
hsa04064	NF-kappa B signaling pathway	*CCL21/CCL19*	2
hsa04062	Chemokine signaling pathway	*CCL21/CCL19*	2
hsa05205	Proteoglycans in cancer	*FLNC/MET*	2
hsa05208	Chemical carcinogenesis—reactive oxygen species	*COX4I2/MET*	2
hsa04060	Cytokine-cytokine receptor interaction	*CCL21/CCL19*	2
hsa04151	PI3K-Akt signaling pathway	*LAMA3/MET*	2
hsa04080	Neuroactive ligand–receptor interaction	*ADCYAP1R1/SSTR1*	2

**Table 3:** Drug combinations with the top synergistic scores (syn_score > 8).

drugA_Name	drug_B_Name	Predicted Synergy Score	Available Reference
Temsirolimus	Nitazoxanide	13.28	/
Temsirolimus	Fisetin	11.86	/
* Cantharidin	* Fisetin	10.35	Frezzato et al. [24]
Fisetin	Enoxacin	9.89	/
* Cantharidin	* ABT-737	9.70	Ren et al. [14]
* Fisetin	* Azithromycin	9.64	Shao et al. [25]
Panobinostat	Cantharidin	9.62	/
Temsirolimus	Rotenone	9.60	/
Temsirolimus	Azithromycin	9.14	/
Cantharidin	Enoxacin	8.32	/

The “*” symbol in the table highlights the drug combinations verified by experimental studies.

**Table 4:** Senolytics and their associated signaling pathways.

Compounds & Signaling Pathway	Description	Reference
Temsirolimus		
PI3K-Akt signaling pathway	Inhibits the proliferation and survival of cancer cells by blocking the PI3K/Akt/mTOR signaling pathway through mTOR inhibition, associated with the core differential genes LAMA3/MET.	Are et al. [27]
mTOR signaling pathway	Directly acts on the mTOR signaling pathway, inhibiting the activity of mTORC1 and mTORC2, thereby inhibiting tumor cell growth and proliferation.	Dancey et al. [28]
Nitazoxanide		
MAPK signaling pathway	κ receptor-induced p38 MAPK phosphorylation mediates restlessness and anxiety in animals, unrelated to analgesic effects, and is mediated by the β-arrestin2 pathway [29].	Khan et al. [29]
PI3K-Akt signaling pathway	Inhibition of mTOR pathway activation can eliminate κ receptor-induced conditioned place aversion (CPA), distinguishing varying degrees of restlessness and anxiety caused by these agonists.	Fan et al. [30]
Neuroactive ligand–receptor interaction	As an opioid receptor agonist-antagonist, involves the interaction of neuroactive ligands with opioid receptors in its analgesic effect.	Cui et al. [31]
Fisetin		
PI3K-Akt signaling pathway	Inhibit the PI3K/AKT signaling pathway, thereby inhibiting mTOR and inducing cell death.	Sun et al. [32]
MAPK signaling pathway	Upregulates HO-1 expression via the p38 MAPK pathway, inhibiting doxorubicin-induced senescence of pulmonary artery endothelial cells.	Kashyap et al. [33]
Nrf2/HO-1 signaling pathway	Inhibits doxorubicin-induced senescence of pulmonary artery endothelial cells and inhibits the proliferation of pulmonary artery smooth muscle cells, thereby preventing pulmonary artery remodeling.	Zhang et al. [34]
Azithromycin		
NF-kappa B signaling pathway	Mitigates inflammatory responses by suppressing the NF-κB signaling pathway.	Xu et al. [35]
Panobinostat		
cAMP signaling pathway	May indirectly affect the cAMP signaling pathway by inhibiting HDAC activity, as HDAC inhibitors can affect multiple cellular signaling pathways, including the cAMP signaling pathway.	Zaccolo et al. [36]
Apoptosis signaling pathway	Increases the acetylation of histones and tubulins, leading to cell cycle arrest and apoptosis by inhibiting HDACs.	Jia et al. [37]
Cell cycle signaling pathway	Induces cell cycle arrest by increasing the level of p21 cell cycle protein.	Prystowsky et al. [38]
Wnt/β-catenin signaling pathway	Inhibits the Wnt/β-catenin signaling pathway by upregulating the expression of APCL.	Qin et al. [39]
JAK2/STAT3 signaling pathway	Inhibits the JAK2/STAT3 signaling pathway in multiple myeloma.	Perrone et al. [40]
Rotenone		
Calcium signaling pathway	Elevates intracellular free calcium ion levels ([Ca^2+^]i) and activates CaMKII, leading to the inhibition of mTOR signaling and the induction of neuronal apoptosis.	Liu et al. [41]
Apoptosis signaling pathway	Induces the production of reactive oxygen species (ROS) in neuronal cells and leads to neuronal apoptosis by inhibiting the mTOR-mediated S6K1 and 4E-BP1 pathways.	Li et al. [42]
mTOR signaling pathway	Induces ROS/H_2_O_2_ to inhibit the mTOR signaling pathway, leading to neuronal apoptosis.	Liu et al. [41]
JAK/STAT3 signaling pathway	Influences the proliferation and apoptosis of oral squamous cell carcinoma cells by regulating the JAK/STAT3 pathway.	Chen et al. [43]
Chemical carcinogenesis—reactive oxygen species	Causes mitochondrial dysfunction, increases the generation of ROS, and results in oxidative damage to proteins, lipids, and nucleic acids.	Li et al. [42]

**Table 5:** Results of molecular docking experiment between nitazoxanide and mTOR.

Mode	Affinity (kcal/mol)	RMSD l.b.	RMSD u.b.
1	−7.259	0	0
2	−6.705	22.38	23.67
3	−6.652	22.23	23.29
4	−6.494	3.092	8.357
5	−6.484	15.39	17.38
6	−6.461	15.81	18.06
7	−6.417	15.16	17.87
8	−6.395	14.52	16.83
9	−6.386	18.74	20.63
10	−6.386	16.06	18.75

**Table 6:** Single senolytic summary sheet (till 1 July 2024).

Drug_Name	Proposed/Known Target(s)	Source
Azacyclonol	Histamine	Patent US 2020/0121620 [49]
Cyclosporin A	Calcineurin, NFAT	Patent US 2020/0121620 [49]
Digoxin	Na^+^/K^+^-ATPase	Triana et al., 2019 [50]
Nitrofural	ROS generation	Patent US 2020/0121620 [49]
Roxithromycin	Protein homeostasis	Ozsvari et al., 2018 [51]
Luteolin	PI3K/Akt, Nrf2, NF-κB	Yousefzadeh et al., 2018 [52]
Enoxacin	TRBP	Patent US 2020/0121620 [49]
Atorvastatin	HMG-CoA, Rho/ROCK	Patent US 2020/0121620 [49]
Azithromycin	Mitochondrial translation	Ozsvari et al., 2018 [51]
Nitazoxanide	phosphorylation,	Patent US 2020/0121620 [49]
Adapalene	RAR/RXR nuclear receptors	Patent US 2020/0121620 [49]
Amiloride hydrochloride	NHE1, ENaC	Triana et al., 2019 [50]
Cantharidin	PP2A	Patent US 2020/0121620 [49]
Calmidazolium chloride	Calmodulin	Guerrero et al., 2019 [53]
Dequalinium chloride hydrate	Mitochondria membrane potential	Patent US 2020/0121620 [49]
Diphenyleneiodonium chloride	NADPH oxidase, flavoproteins	Patent US 2020/0121620 [49]
2,3-Dimethoxy-1,4-naphthoquinone	Redox cycling	Patent US 2020/0121620 [49]
Idarubicin	Topoisomerase II	Patent US 2020/0121620 [49]
JFD00244	SIRT6	Guerrero et al., 2019 [53]
Mibefradil dihydrochloride	T-type calcium channels	Guerrero et al., 2019 [53]
Piperlongumine	TrxR/GPx	Wang et al., 2016 [54]
Ouabain	Na^+^/K^+^-ATPase	Guerrero et al., 2019 [53]
Quercetin dihydrate	PI3K, HSP90, AMPK, Nrf2	Zhu et al., 2015 [55]
Rottlerin	PKCδ	Guerrero et al., 2019 [53]
Rotenone	Complex I (ETC)	Guerrero et al., 2019 [53]
BIX 01294 trihydrochloride hydrate	G9a/GLP (EHMT2/1)	Guerrero et al., 2019 [53]
Tyrphostin AG 879	ErbB2, TrkA	Patent US 2020/0121620 [49]
Vincristine sulfate	Tubulin	Patent US 2020/0121620 [49]
Tanespimycin	HSP90	Fuhrmann-Stroissnigg et al., 2017 [56]
Geldanamycin	HSP90	Fuhrmann-Stroissnigg et al., 2017 [56]
Alvespimycin	HSP90	Fuhrmann-Stroissnigg et al., 2017 [56]
ProDrug A	unknown	Guerrero et al., 2020 [57]
JHB76B	KRAS/ERK pathway	Guerrero et al., 2020 [57]
CGP-74514A	CDK1/2	Guerrero et al., 2019 [53]
Ouabagenin	Na^+^/K^+^-ATPase	Guerrero et al., 2019 [53]
K-Strophanthin	Na^+^/K^+^-ATPase	Guerrero et al., 2019 [53]
Strophanthidin	Na^+^/K^+^-ATPase	Guerrero et al., 2019 [53]
PF-573228	FAK	Patent US 2020/0121620 [49]
LY-367265	5-HT1B/1D	Patent US 2020/0121620 [49]
Temsirolimus	mTORC1	Patent US 2020/0121620 [49]
Eltrombopag	MPL (TPO -R)	Patent US 2020/0121620 [49]
Raltegravir	HIV integrase	Patent US 2020/0121620 [49]
Venetoclax	BCL-2	Lafontaine et al., 2021 [58]
EF24	NF-κB/IκB-α	Li et al., 2019 [59]
Panobinostat	HDAC	Samaraweera et al., 2017 [60]
Bufalin	Na^+^/K^+^-ATPase	Triana et al., 2019 [50]
Proscillaridin A	Na^+^/K^+^-ATPase	Triana et al., 2019 [50]
Cinobufagin	Na^+^/K^+^-ATPase	Triana et al., 2019 [50]
Peruvoside	Na^+^/K^+^-ATPase	Triana et al., 2019 [50]
Digitoxin	Na^+^/K^+^-ATPase	Triana et al., 2019 [50]
Convallotoxin	Na^+^/K^+^-ATPase	Triana et al., 2019 [50]
ABT-737	BCL-2, BCL-xL, BCL-w	Yosef et al., 2016 [61]
Fisetin	PI3K, NF-κB, HIF-1α, Nrf2	Yousefzadeh et al., 2018 [52]
Curcumin	NF-κB, Nrf2, HAT/HDAC	Yousefzadeh et al., 2018 [52]
Dasatinib	SRC/ABL kinases	Zhu et al., 2015 [55]
Navitoclax	BCL-2, BCL-xL	Zhu et al., 2016 [62]
A1331852	BCL-xL	Zhu et al., 2017 [56]
A1155463	BCL-xL	Zhu et al., 2017 [56]
ginkgetin	JAK/STAT, NF-κB	Smer-Barreto et al., 2023 [48]
oleandrin	Na^+^/K^+^-ATPase	Smer-Barreto et al., 2023 [48]
periplocin	Na^+^/K^+^-ATPase	Smer-Barreto et al., 2023 [48]
BRD-K56819078	HSP90	Wong et al., 2023 [63]
XL888	HSP90	Wong et al., 2023 [63]

## Data Availability

The original contributions presented in this study are included in the article/Supplementary Material. Further inquiries can be directed to the corresponding author.

## Supplementary Materials

The following supporting information can be downloaded at: https://www.mdpi.com/article/10.3390/ijms26189004/s1.
